# Efficiency and equity of resource allocation in healthcare services using DEA and concentration indices: evidence from the traditional medicine hospital in Gansu Province, China

**DOI:** 10.3389/fpubh.2025.1674348

**Published:** 2025-10-01

**Authors:** Yahui Ba, Zhonghua Luo

**Affiliations:** ^1^School of Health Management, Gansu University of Chinese Medicine, Lanzhou, Gansu, China; ^2^School of Marxism, Gansu University of Chinese Medicine, Lanzhou, Gansu, China

**Keywords:** TCM hospital, efficiency, concentration indices, DEA, resources, health inequalities

## Abstract

**Background:**

Traditional Chinese medicine (TCM) hospitals are vital to China's healthcare system, yet concerns persist about the efficiency and equity of resource allocation. Variations in economic development across cities within a province lead to disparities in allocation efficiency, with many TCM hospitals facing challenges like low technical efficiency and uneven resource distribution. These issues hinder quality healthcare delivery and compromise fairness.

**Methods:**

Using health statistics data (2018–2024) from China's Gansu Provincial Bureau of Statistics, this study employed a three-stage Data Envelopment Analysis (DEA) model, the Malmquist index, and Health Resources Agglomeration Degree (HRAD) to evaluate the efficiency and equity of TCM hospital resource allocation across 14 cities in the province.

**Results:**

In 2024, the comprehensive technical efficiency of healthcare services at Gansu Provincial TCM Hospital was 0.961, with 10 regions being DEA-effective, 1 showing weak effectiveness, and 3 deemed ineffective. After adjusting environmental variables and random disturbances in the third-stage analysis, the recalculated efficiency metric stands at 0.962, showing minimal variation. This indicates that environmental factors exert a negligible influence on efficiency. From 2018 to 2024, total factor productivity declined annually, indicating significant room for efficiency improvement. Resource allocation equity varied widely across regions, with notable disparities in both geographical concentration and population-based distribution observed during the study period.

**Conclusions:**

The efficiency and equity of resource allocation of TCM hospitals require substantial improvement. Insufficient resources limit hospital performance, and while technical efficiency surpasses allocation efficiency, overall technical standards remain inadequate. Geographic inequities in resource distribution are particularly pronounced. To address these challenges, establishing a provincial resource allocation mechanism, enhancing infrastructure in low-efficiency areas, and coordinating resource distribution across economically diverse cities are essential to optimize both efficiency and fairness.

## 1 Introduction

This study aims to evaluate the technical efficiency and equity of resource allocation in Traditional Chinese Medicine (TCM) hospitals across Gansu Province, and to propose targeted strategies for improvement. The efficiency of resource allocation in healthcare services refers to the optimal use and distribution of resources to provide healthcare services and achieve expected health outcomes (1, 2). In the context of sustainable development (3), equitable allocation of resources for healthcare services is crucial for addressing public health issues. However, differing levels of economic development significantly influence the fairness of resource allocation. As one of the most populous countries in the world, China's healthcare needs and coverage scale are of significant global significance. China needs to place greater emphasis on the fairness of healthcare resource allocation. Traditional Chinese medicine hospitals are crucial to China's healthcare system, but their resource allocation efficiency and fairness remain concerning. Within the same province, cities with different levels of economic development exhibit varying resource allocation efficiencies (4). Many traditional Chinese medicine hospitals face challenges such as low technical efficiency and unequal resource distribution, which may hinder the provision of high-quality healthcare services and result in low fairness.

Current scholars have conducted research on traditional Chinese medicine (TCM) hospitals from various perspectives, including compensation mechanisms, resource allocation, and operational efficiency. Deng Weifeng and other scholars analyzed the compensation mechanisms of county-level TCM hospitals in China (5). Liu Yuzhuo and other scholars studied the fairness of health resource allocation in specific provinces of China (6). Li Jing and other scholars analyzed the efficiency of health resource allocation in different types of TCM hospitals (7). Zang Zitong and other scholars researched the efficiency of medical services in township health centers. Research on various aspects of TCM hospitals is relatively abundant (8), yet studies specifically targeting the efficiency and fairness of resource allocation for medical and health services in TCM hospitals remain scarce. Additionally, existing research on the efficiency and fairness of resource allocation for medical and health services in TCM hospitals primarily focuses on the macro level (9–13), with the selected scope often centered on inequality issues at the national and global levels, while neglecting the micro level. Studies targeting regional, urban, and community levels are relatively limited (14). By studying cities within the same province that have significant disparities in various aspects, it is possible to provide a reference for other countries and regions with large economic disparities or poor overall economic conditions. As a representative city of China's western region (i.e., a relatively underdeveloped region), Gansu exhibits significant disparities in economic development among its cities, which can effectively reflect the interplay between health and inequality in a sustainable context. Against this backdrop, this paper takes the 14 cities within Gansu Province as its research subjects, analyzing the static efficiency of healthcare resources in traditional Chinese medicine hospitals across Gansu Province, changes in dynamic efficiency, and the fairness of resource allocation. Based on these findings, this study aims to draw on existing case studies to provide more targeted recommendations for economically underdeveloped regions in western China, as well as other economically lagging countries and regions. This will help to more effectively alleviate health inequalities and enhance the fairness of healthcare services.

In the healthcare sector, traditional data envelopment analysis (DEA) or Malmquist models are commonly used (15, 16), which only calculate efficiency values (17, 18). This paper focuses on discussing the efficiency and inequality of healthcare resource allocation among cities with different levels of economic development within the same province in the context of sustainable development, providing reference for China and other countries in improving the efficiency and fairness of healthcare resource allocation. Therefore, this paper uses a combination of the three-stage DEA model, the Malmquist index model, and the health resources agglomeration degree (HRAD) model. Using a three-stage DEA model, the first stage employs the traditional DEA-BCC model for calculation. In the second stage, the Stochastic Frontier Analysis (SFA) model is used to eliminate the influence of environmental factors such as the economy and population. Finally, in the third stage, the data is recalculated using DEA after adjusting for management inefficiencies, thereby eliminating the impact of socioeconomic factors on the allocation and equity of healthcare resources. Combining the three-stage DEA model with the Malmquist model not only enables horizontal static calculations but also vertical dynamic calculations across different years, thereby broadening the scope of the research. The introduction of the HRAD method allows this study to further explore fairness while discussing efficiency. Therefore, based on the latest statistical data from the official website of the Gansu Provincial Government from 2018 to 2024, this study conducts a multi-angle analysis of the resource allocation efficiency and fairness of traditional Chinese medicine hospitals in 14 cities in Gansu Province. By examining the resource allocation efficiency and inequality of healthcare services in different economic regions under the context of sustainable development, this study aims to reveal the characteristics and shortcomings of current policies, thereby providing theoretical support and practical references for China and other countries in enhancing the efficiency and equity of healthcare service resource allocation.

The innovations and contributions of this study are as follows: First, the scope of the study was narrowed down to cities, with cities in Gansu selected for analysis, enriching the research results on healthcare services at the micro level and effectively improving the situation where research at the macro level is extensive but research at the micro level is insufficient. Second, the study combines the three-stage DEA model with the Malmquist model, which not only enables horizontal static measurement through the DEA model, but also enables vertical dynamic measurement across different years through the Malmquist model, reflecting trends in production efficiency over time and broadening the scope of the study. Third, combining the DEA three-stage model with the HRAD analysis method not only enables research on efficiency but also covers research on fairness, thereby achieving innovation in research methods. Fourth, the second stage of the DEA three-stage model involves constructing a SFA model that eliminates the influence of external factors, such as environmental variables and random disturbances. Finally, this study provides valuable experience and reference for future research and other regions to achieve high-quality and coordinated healthcare development.

## 2 Materials and methods

### 2.1 Data sources

The data for this study was sourced from the Gansu Provincial Bureau of Statistics' official website, specifically from the “Gansu Province Traditional Chinese Medicine Development Statistics Bulletin” and the “Gansu Province Health and Wellness Development Statistics Bulletin.” Taking into account the timeliness and availability of the data, and ensuring that the time frame was as comprehensive as possible, the period from 2018 to 2022 was ultimately selected for data analysis. This study examined the efficiency and equity of healthcare service distribution in 14 cities in Gansu Province. In the literature on healthcare efficiency, commonly used indicators typically include physical resources, financial resources, and human resources (19), while output indicators typically reflect service utilization rates and performance outcomes (20, 21). Through a comprehensive review of the literature and in consultation with knowledgeable experts, seven input-output indicators that effectively reflect resource allocation efficiency were selected, taking into consideration the rationality, authenticity, and availability of the indicator data. The input indicators include traditional Chinese medicine (TCM) healthcare human resources, the number of TCM diagnostic and therapeutic devices (valued at over 5,000 yuan), the number of TCM healthcare institutions, and the number of TCM beds. The output indicators include the number of TCM prescriptions, the number of TCM consultations, and the number of discharges from TCM medical institutions. At the same time, since socioeconomic development has a significant impact on the efficiency and fairness of resource allocation, and considering differences in socioeconomic factors such as economic level, healthcare status, and population density, we selected three indicators—per capita GDP, permanent resident population (in ten thousand), and the proportion of traditional Chinese medicine (TCM) outpatient visits to the total number of outpatient visits in the region—as independent variables, as shown in Table 1.

**Table 1:** Input-output indicators.

**Type of indicator**	**Name of indicator**	**Observations**	**Mean**	**Std. dev**.	**Max**	**Min**
Input indicators	TCM healthcare human resources (units)	98	1,468.980	876.752	5,052.000	195.000
	Number of TCM diagnostic and therapeutic devices (more than 5,000 yuan) (units)	98	474.878	397.170	2,233.000	8.000
	Number of TCM healthcare institutions (units)	98	127.786	80.446	555.000	3.000
	Number of TCM beds (units)	98	3,333.418	1,972.546	7,768.000	482.000
Output indicators	Number of TCM prescriptions (units)	98	1,795,891.500	1,078,139.922	6,056,365.000	87,506.000
	Number of TCM consultations (10,000)	98	161.184	90.573	470.550	27.400
	Number of hospital discharges from TCM medical institutions (person)	98	88,402.000	57,473.290	249,708.000	9,836.000
Independent variable	Per capita GDP (CNY)	98	48,092.415	31,379.621	143,784.000	12,447.000
	Permanent resident population (10,000)	98	180.530	105.382	443.650	25.200
	The proportion of TCM outpatient visits to the total number of outpatient visits in the region (percentage)	98	11.996	9.769	28.710	0.210

### 2.2 Research methods

#### 2.2.1 Data envelopment analysis model

DEA is a method based on relative efficiency evaluation. By calculating the relative efficiency of decision-making units (DMUs), it determines the effectiveness of DMUs (22–25). If a DMU is found to be DEA-effective, it indicates that it has achieved optimal efficiency. If a DMU is found to be DEA-ineffective, it means that its efficiency has not reached the optimal level, and adjustments to inputs and outputs are needed to improve efficiency. Slack variables in data envelopment analysis refer to the difference between actual inputs and ideal inputs under the same output conditions. They reflect the degree of inefficiency in inputs for decision-making units. When DEA is ineffective, slack variables (S– and S+) measure input excess or output deficiency in decision units, revealing efficiency losses and guiding efficiency improvements. The input slack variable (S–) indicates the amount of input that a decision unit can reduce without affecting output at the current output level. The output slack variable (S+) indicates the amount of output that the decision-making unit can increase at the current input level. If S– is greater than 0, it indicates that the decision-making unit has excess inputs. If S+ is greater than 0, it indicates that there is insufficient output. When S– or S+ equals 0, the output-to-input ratio reaches the optimal level, achieving the effectiveness of DEA. The ideal value in data envelopment analysis refers to the maximum output that can be achieved given a certain input level, or the minimum input that can be achieved given a certain output level, representing the theoretical production level on the efficiency frontier. Data envelopment analysis is not limited by the number of input or output indicators and identifies the optimal input-output scheme, primarily including the Banker Charnes Cooper(BCC) model, Charnes Cooper Rhodes(CCR) model, and Malmquist index model, among others (26). In the DEA model, the indicator reflecting resource allocation efficiency is comprehensive technical efficiency. The three-stage model evolved from the basic DEA model. The three-stage DEA model consists of the following three steps:

(1) First stage: traditional DEA method

Traditional DEA models mainly include two types: one is the CCR model (Charnes Cooper Rhodes), which assumes constant returns to scale. This model assumes that inputs and outputs must increase or decrease in equal proportions, an idealized scenario that is difficult to achieve in reality. The other is the BCC model (Banker Charnes Cooper), which takes into account the possibility of changes in scale efficiency in actual production. This model allows for variable returns to scale, which is closer to reality. Due to the overly stringent assumptions of the CCR model, scholars have made improvements based on it, thereby developing the more flexible and practical BCC model (27). The DEA-BCC model calculates efficiency values from a horizontal static perspective, dividing the evaluated efficiency into three types: comprehensive efficiency, pure technical efficiency, and scale efficiency. Technical efficiency (TE) consists of pure technical efficiency (PTE) and scale efficiency (SE). In data envelopment analysis, technical efficiency refers to the technical efficiency of a decision-making unit at its optimal scale. It can be decomposed into pure technical efficiency, i.e., technical efficiency without considering scale effects and scale efficiency, i.e., the efficiency difference between the actual scale and the optimal scale (28–33). As shown in Equation 1:


(1) $$\begin{array}{l} \;\min\left[\theta -\varepsilon \left({\text{e}}^{\text{T}}{\text{s}}^{-}+{\text{e}}^{\text{T}}{\text{s}}^{+}\right)\right]\\ \text{ s}.\text{t}.\sum_{\text{j=1}}^{\text{n}}{\text{x}}_{\text{j}}\lambda_{\text{j}}+{\text{s}}^{-}=\theta {\text{x}}_{0}\\ \;\sum_{\text{j=1}}^{\text{n}}{\text{y}}_{\text{j}}\lambda_{\text{j}}-{\text{s}}^{+}={\text{y}}_{0}\\ \sum_{\text{j=1}}^{\text{n}}\lambda_{\text{j}}=1\\ \lambda_{\text{j}}\geq 0,\text{j=1},\ldots ,\text{n}.\\ {\text{s}}^{+}\geq 0,{\text{s}}^{-}\geq 0 \end{array}$$

θ denotes the technical efficiency value of the jth decision-making unit (DMU) (0 <θ ≤ 1). When θ = 1, the DMU is efficient; when θ <1, it is inefficient. ε denotes the Archimedean infinitesimal, used to ensure the existence of slack variables (s-, s+) and prevent the objective function from degenerating into a simple θ_j_ calculation. s- represents the input slack variable, indicating the excess amount required when the DMU's actual input exceeds the effective frontier (s-≥0). s+ is the output slack variable, representing the shortfall between the DMU's actual output and the effective demand (s+≥0). e^T^ denotes the transpose of the unit vector, such as e=[1,1,...,1]^T^, used to aggregate the sums of slack variables (e^T^s^−^ represents the sum of input slack, while e^T^s^+^ denotes the sum of output slack).

The comprehensive technical efficiency (TE) measured by the BCC model can be decomposed into the product of pure technical efficiency (PTE) and scale efficiency (SE). As shown in Equation 2:


(2) $$\begin{array}{c} TE=PTE\times SE \end{array}$$

Scale returns are categorized into three states: increasing returns to scale (IRS), constant returns to scale (CRS), and decreasing returns to scale (DRS). These are measured based on inputs to assess pure technical and scale efficiency, thereby demonstrating the relative effectiveness of decision-making units and their scale return states. When a decision-making unit is DEA-efficient (*TE* = 1), it typically means that it has achieved technical efficiency (TE), indicating that the input-output balance of the evaluation unit is optimal. This means that the decision-making unit's pure technical efficiency and scale efficiency in resource utilization are both optimal at the current scale. DEA weak efficiency typically refers to a decision-making unit that is not optimal in certain aspects, possibly with room for improvement in terms of pure technical efficiency or scale efficiency, but is at least efficient in one aspect (such as pure technical efficiency). If TE, PTE, and SE are all less than 1, it indicates that the decision-making unit is “inefficient,” with an imbalance between inputs and outputs. When returns to scale are increasing, inputs should be increased to expand outputs; when returns to scale are decreasing, inputs should be stabilized to improve efficiency; when returns to scale are constant, DEA is efficient.

Although traditional DEA can intuitively evaluate resource utilization efficiency (34), Fried believes that the performance of decision-making units is affected by external environments and management inefficiencies. Therefore, it is necessary to use the SFA method to separate these influences.

(1) Second stage: SFA method

Aigner first proposed the SFA to estimate the production efficiency of organizations, identify random factors in the production process, and decompose random factors to obtain random errors, thereby eliminating the impact of random errors on efficiency (35).

First, calculate the slack variables for each decision unit in the first stage. Their values are equal to the actual input values of each decision unit minus the input target values corresponding to the DEA model, thereby obtaining the slack quantities for each input item. Next, use these calculated slack variables as the dependent variables and the selected environmental variables as the independent variables to construct a Stochastic Frontier Analysis (SFA) regression model for regression analysis. The basic functional form of the SFA regression model is as shown in Equation 3 and Equation 4:


(3) $$\begin{array}{ccc} S_{ni} & = & f\left(Z_{i}\beta_{n}\right)+v_{ni}+\mu_{ni} \end{array}$$

(4) $$\begin{array}{ccc} \text{E}\left[{\text{v}}_{\text{ni}}+\mu_{\text{ni}}\right] & = & {\text{s}}_{\text{ni}}-\text{f}\left({\text{z}}_{\text{i}},\beta_{\text{n}}\right)-\text{E}\left[{\text{u}}_{\text{ni}}|{\text{v}}_{\text{ni}}+\mu_{\text{ni}}\right] \end{array}$$

S_ni_ is the slack amount (n = 1, 2, …, n; i = 1, 2, …, n)of the n-th input of the i-th decision making unit, Z_i_(i = 1.2,…, I) is the i-th environmental variable, β_n_(n = 1, 2, …, N) is the environmental measurement coefficient of the n-th decision unit, μ_ni_ denotes the measurement error term, u_ni_ denotes the technical inefficiency term, and v_ni_ is a random error following a normal distribution.

After excluding the impact of environmental factors and random errors on the analysis efficiency through regression models, the adjusted input variables are calculated using the Equation 5:


(5) $$\begin{array}{l} X_{ni}^{A}=X_{ni}+[\max(f(Z_{i};{\hat{\beta }}_{n}))-f(Z_{i};{\hat{\beta }}_{n})]+[\max(\nu_{ni})-\nu_{ni}]\\ \;i=1,2,\cdots ,I;n=1,2,\cdots ,N \end{array}$$

Among them, $X_{ni}^{A}$ is the input amount in the first stage; X_ni_ is the adjusted input; $\left[\max\left(f\left(Z_{i};{\hat{\beta }}_{n}\right)\right)-f\left(Z_{i};{\hat{\beta }}_{n}\right)\right]$ is the adjustment for external environmental factors; [max(v_ni_) -v_ni_] is the same state level after excluding random errors from all decision units (36).

(3) Third stage: adjusted DEA method

After eliminating the interference of environmental factors and random errors in the second stage, the adjusted input quantity and output quantity from the first stage are re-entered into the traditional DEA model to obtain new efficiency values that are more objective and accurate.

#### 2.2.2 Malmquist model

The Malmquist index model is a dynamic method for measuring production efficiency proposed by Swedish economist Sten Malmquist in 1953 (37). Its core principle is to construct a productivity index using a distance function ratio, thereby overcoming the limitations of traditional static efficiency measurement methods. From a longitudinal dynamic perspective, the efficiency changes of traditional Chinese medicine hospitals are evaluated by calculating the total factor productivity index (TFP), which is commonly used to analyze panel data and can illustrate the efficiency changes and trends of decision-making units. In the Malnquist model, the indicator reflecting resource allocation efficiency is total factor productivity. Total factor productivity is composed of the technical efficiency index (TC), pure technical efficiency change (PEC), and scale efficiency change (SEC). The technical progress change (TC) index reflects changes in the level of technological progress. The technical efficiency change (EC) index reflects changes in resource utilization efficiency, including the pure efficiency change (PECT) index and the scale efficiency change (SEC) index (21). As shown in Equation 6:


(6) $$\begin{array}{l} TFPch=M(x_{t+1},y_{t+1},x_{t},y_{t})\\ \;=[\frac{D_{t}(x_{t+1},y_{t+1})}{D_{t}(x_{t},y_{t})}\times \frac{D_{t+1}(x_{t+1},y_{t+1})}{D_{t+1}(x_{t},y_{t})}{]}^{\frac{1}{2}}\\ \;Effch=\frac{D_{t}\left(x_{t+1},y_{t+1}\right)}{D_{t}\left(x_{t},y_{t}\right)}\\ TEch={\left[\frac{D_{t}\left(x_{t+1},y_{t+1}\right)}{D_{t+1}\left(x_{t+1},y_{t+1}\right)}\times \frac{D_{t}\left(x_{t},y_{t}\right)}{D_{t+1}\left(x_{t},y_{t}\right)}\right]}^{\frac{1}{2}} \end{array}$$

X_t_ denotes the input vector for period t, while y_t_ denotes the output vector for period t. X_t+1_ denotes the input vector for period t+1, while y_t+1_ denotes the output vector for period t+1.

D_t_(x, y) denotes the distance function under the technology at time t, measuring the minimum input x required to achieve a given output y. D_t+1_(x, y)) denotes the distance function under the technology at time t+1, measuring the minimum input x required to achieve a given output y.

The specific calculation formula is shown in Equation 7:


(7) $$\begin{array}{c} TFP=TC\times EC=TC\times PECT\times SEC \end{array}$$

If all index values are 1, it indicates that efficiency is stabilizing. Values greater than 1 or less than 1 indicate that efficiency is trending upward or downward, respectively.

#### 2.2.3 Health resources agglomeration degree

Health resources agglomeration degree (HRAD) refers to the degree of concentration of certain resources or population elements in a given region, primarily reflecting the spatial distribution of these elements. Specifically, it measures the concentration of production factors within a specific region relative to their concentration in the higher-level region. The level of HRAD impacts regional economic development, resource allocation efficiency, and competitiveness (38, 39). is As shown in Equation 8:


(8) $$\begin{array}{c} HRAD=\frac{\frac{HR_{i}}{HR_{n}}*100\%}{\frac{A_{i}}{A_{n}}*100\%}=\frac{\frac{HR_{i}}{A_{i}}}{\frac{HR_{n}}{A_{n}}} \end{array}$$

HR_i_ represents the total amount of health resources available in the i-th city, and A_i_ denotes its corresponding land area; HR_n_ represents the total amount of health resources in the higher-level administrative region (e.g., province) to which the city belongs, and A_n_ denotes the total land area of that higher-level region. The HRAD index is used to measure the fairness of the distribution of health resources across geographic areas: when HRAD equals 1, it indicates that resources are absolutely evenly distributed across geographic areas; if HRAD is greater than 1, it suggests that health resource allocation is relatively balanced or there is a relative surplus of resources relative to land area; if HRAD is less than 1, it indicates that resource allocation is unevenly distributed across geographic areas, and fairness needs to be improved (40).

Population Agglomeration Degree (PAD) reflects the ratio of the population per square kilometer of land area to the population per square kilometer of land area in the higher-level region, analyzing the fairness of population distribution (41, 42). The calculation formula is as Equation 9:


(9) $$\begin{array}{c} PAD_{i}=\frac{\frac{p_{i}}{p_{n}}\times 100\%}{\frac{A_{i}}{A_{n}}\times 100\%} \end{array}$$

Where P_i_ refers to the population of city i, P_n_ refers to the total population of the province to which the city belongs, A_i_ refers to the land area of city i, and A_n_ refers to the total land area of the province to which the city belongs.

The ratio of health resource concentration to population concentration (i.e., HRAD/PAD) reflects the fairness of health resource allocation based on population size. As shown in Equation 10:


(10) $$\begin{array}{c} \frac{HRAD}{PAD}=\frac{\frac{HR_{i}}{HR_{n}}}{\frac{P_{i}}{P_{n}}} \end{array}$$

Where P_i_ refers to the population of city i, P_n_ refers to the total population of a certain region, A_i_ refers to the land area of city i, and A_n_ refers to the total land area of a certain region. If $\frac{HRAD_{\text{i}}}{PAD_{i}}=1$, resources are basically sufficient to meet the needs of residents in the agglomeration area. When the ratio is greater than 1, resources are relatively abundant, and when the ratio is less than 1, resources are relatively scarce.

HR_i_ represents the health resources available in each city, HR_n_ represents the total health resources of the corresponding higher-level region (provincial level), P_i_ represents the total population of each city, and P_n_ represents the total population of the corresponding provincial level. A ratio of HRAD to PAD equal to 1 indicates that health resources are sufficient to meet the needs of residents in the aggregated region; a ratio greater than 1 indicates relative resource surplus, with higher fairness in the distribution of health resources according to population size; a ratio less than 1 indicates relative resource shortage, with lower fairness in the distribution of health resources according to population size. The data for this method in this study primarily originate from the “Gansu Province Health and Wellness Development Statistical Bulletin” published on the official website of the Gansu Provincial Bureau of Statistics.

## 3 Results

### 3.1 Results of DEA model

#### 3.1.1 Results of the three-stage DEA model

(1) Phase I: Results of the traditional DEA model

The traditional DEA-BCC model was used to analyze the static efficiency of resource allocation for medical and health services in TCM hospitals in Gansu Province in 2024. The results are shown in Table 2.

**Table 2:** Results of traditional DEA analysis.

**Zones**	**Comprehensive technical efficiency (TE)**	**Pure technical efficiency (PTE)**	**Scale efficiency (SE)**	**Scale returns**	**Relative efficiency**
Lanzhou city (LZ)	1.000	1.000	1.000	–	Efficiently
Jiayuguan city (JYG)	1.000	1.000	1.000	–	Efficiently
Jinchang city (JC)	1.000	1.000	1.000	–	Efficiently
Baiyin city (BY)	0.962	0.975	0.987	irs	Ineffectively
Tianshui city (TS)	1.000	1.000	1.000	–	Efficiently
Wuwei city (WW)	1.000	1.000	1.000	–	Efficiently
Zhangye city (ZY)	0.975	1.000	0.975	drs	Weakly effective
Pingliang city (PL)	0.908	0.919	0.988	irs	Ineffectively
Jiuquan city (JQ)	1.000	1.000	1.000	–	Efficiently
Qingyang city (QY)	1.000	1.000	1.000	–	Efficiently
Dingxi city (DX)	1.000	1.000	1.000	–	Efficiently
Longnan city (LN)	1.000	1.000	1.000	–	Efficiently
Linxia state (LX)	1.000	1.000	1.000	–	Efficiently
Gannan state (GN)	0.610	0.695	0.878	irs	Ineffectively
Average value	0.961	0.971	0.988		

As shown in Table 2, the efficiency of TCM hospital resource allocation in 14 cities of Gansu Province in 2024 is as follows: LZ, JYG, JC, TS, WW, JQ, QY, DX, LN, and LX have a comprehensive TE value of 1, achieving DEA efficiency, with TCM resource allocation efficiency meeting standards; ZY only has a PTE value of 1, with DEA being weakly efficient, and TCM resource allocation efficiency basically meeting standards; The TE, PTE, and SE values for the three cities of BY, PL, and GN are all less than 1, indicating DEA inefficiency, and the efficiency of TCM resource allocation does not meet the standards; among them, GN has a TE value of 0.610, indicating the worst efficiency of TCM resource allocation. From the results of scale returns, ZY exhibits decreasing returns (drs), while BY, PL, and GN exhibit increasing returns (irs). The remaining 10 cities show constant scale returns, indicating that decision-making units have basically achieved optimal production efficiency under current resource conditions at their current scale.

Based on the DEA analysis results, a table of slack variables and ideal values for DEA-inefficient cities was compiled, as shown in Tables 3, 4. As can be seen from the table, among the input indicators of the three DEA-inefficient cities, three slack variables (S2-, S3-, and S4-) for BY and PL are 0.000, reaching the optimal level. For GN, while S4- is 0.000 and has reached the optimal level, none of the other indicators have reached the optimal level. Among the output indicators, GN has only one variable (S3+) that has reached the optimal level, while BY and PL each have two slack variables that have reached the optimal level. For BY, S2+ and S3+ have reached the optimal level, and for PL, S1+ and S3+ have reached the optimal level. Traditional Chinese medicine hospitals in different regions exhibit significant differences in efficiency due to varying levels of economic development, which result in differences in resource allocation efficiency and fairness.

**Table 3:** Slack variables and ideal values of input indicators for TCM hospitals in DEA invalid cities of Gansu Province in 2024.

**Zones**	**Input indicators**							
	**S1–**	**Ideal value**	**S2–**	**Ideal value**	**S3–**	**Ideal value**	**S4–**	**Ideal value**
BY	191.277	1,325.795	0.000	354.893	0.000	150.147	0.000	2,202.484
PL	102.444	2,163.003	0.000	669.065	0.000	230.680	0.000	4,421.527
GN	67.323	665.166	281.303	161.387	3.026	64.385	0.000	980.590

S1-, S2-, S3-, and S4- represent the slack variables for TCM healthcare human resources, the number of TCM diagnostic and therapeutic devices (valued at over 5,000 yuan), number of TCM healthcare institutions, and the number of TCM beds, respectively.

**Table 4:** Slack variables and ideal values of output indicators for TCM hospitals in DEA invalid cities of Gansu Province in 2024.

**Zones**	**Output indicators**					
	**S1+**	**Ideal value**	**S2+**	**Ideal value**	**S3+**	**Ideal value**
BY	79,693.350	1,441,897.350	0.000	138.560	0.000	64,149.000
PL	0.000	2,303,590.000	14.086	222.806	0.000	138,099.000
GN	204,127.072	578,525.072	8.052	61.522	0.000	26,197.000

S1+, S2+, and S3+ represent the relaxed variables for the number of TCM prescriptions, the number of TCM consultations, and the number of hospital discharges from TCM medical institutions, respectively.

(2) Phase II: SFA regression model results

In the first stage of the DEA analysis, the input slack variable was used as the dependent variable to investigate the impact of environmental variables on it. The environmental variables per capita GDP, permanent resident population (in ten thousand), and the proportion of traditional Chinese medicine (TCM) outpatient visits to the total number of outpatient visits in the region were used as explanatory variables for SFA regression analysis. The sign of the regression coefficient is inversely related to changes in efficiency: a positive regression coefficient indicates that an increase in the variable leads to an increase in the slack variable, resulting in resource waste and reduced efficiency; a negative regression coefficient means that an increase in the variable leads to a decrease in the slack variable, thereby reducing waste and improving efficiency. In the study, per capita GDP and permanent resident population had a significant positive impact on TCM healthcare human resources, the number of TCM diagnostic and treatment devices (worth over 5,000 yuan), the number of TCM healthcare institutions, and the number of TCM beds. However, proportion of TCM outpatient visits to the total number of outpatient visits in the region had a significant negative impact. For details, see Table 5.

**Table 5:** SFA regression results.

**Variable**	**TCM healthcare human resources (units)**	**Number of TCM diagnostic and therapeutic devices (more than 5,000 yuan) (units)**	**Number of TCM healthcare institutions (units)**	**Number of TCM beds (units)**
Constant term	7.69E+01^***^	−3.86E+01^***^	5.16E-01	9.66E+00^***^
Per capita GDP (CNY)	−1.02E-03^***^	−2.88E-04	−8.71E-05	−8.07E-04
Permanent population (10,000)	−4.71E-01^***^	−4.06E-01^*^	−2.51E-02	−4.27E-01
The proportion of TCM patients in the total number of local consultations (percentage)	1.88E+00^**^	4.82E+00^***^	3.11E-01	4.04E+00^***^
σ^2^	3.22E+04^***^	2.94E+04^***^	1.74E+02^***^	4.06E+04^***^
Γ	1.00E+00^***^	1.00E+00^***^	1.00E+00^***^	1.00E+00^***^
LR one-sided test	1.08E+01^**^	1.24E+01^***^	1.09E+01^***^	1.16E+01^***^

^*^indicates *p* < 0.05,^**^ indicates *p* < 0.01, ^***^ indicates *p* < 0.001.

(3) Phase III: analysis results of the adjusted DEA model

A DEA analysis was conducted again on the adjusted data from the second stage, with the results shown in Table 6. The adjusted TE increased from 0.961 to 0.962, the PTE increased from 0.971 to 0.978, and the SE decreased from 0.988 to 0.981. This indicates that the traditional DEA method underestimated the TE and PTE, while overestimating the scale efficiency value. Specifically, the adjusted TE of JC has decreased, indicating that independent variables have a positive effect on it. Meanwhile, the comprehensive efficiency of BY, ZY, PL, and JN has increased, indicating that environmental variables have a negative effect on them. Although the comprehensive efficiency of BY, PL, and GN has improved, it still has not reached an effective level, indicating that the external environment has a certain impact on them. It is necessary to improve the economic level to further enhance comprehensive efficiency.

**Table 6:** Analysis results of adjusted DEA model.

**Zones**	**Comprehensive technical efficiency (TE)**	**Pure technical efficiency (PTE)**	**Scale efficiency (SE)**	**Scale returns**	**Relative efficiency**
Lanzhou city (LZ)	1.000	1.000	1.000	–	Efficiently
Jiayuguan city (JYG)	1.000	1.000	1.000	–	Efficiently
Jinchang city (JC)	0.988	1.000	0.988	irs	Weakly effective
Baiyin city (BY)	0.956	0.997	0.959	irs	Ineffectively
Tianshui city (TS)	1.000	1.000	1.000	–	Efficiently
Wuwei city (WW)	1.000	1.000	1.000	–	Efficiently
Zhangye city (ZY)	1.000	1.000	1.000	–	Efficiently
Pingliang city (PL)	0.911	0.925	0.984	irs	Ineffectively
Jiuquan city (JQ)	1.000	1.000	1.000	–	Efficiently
Qingyang city (QY)	1.000	1.000	1.000	–	Efficiently
Dingxi city (DX)	1.000	1.000	1.000	–	Efficiently
Longnan city (LN)	1.000	1.000	1.000	–	Efficiently
Linxia state (LX)	1.000	1.000	1.000	–	Efficiently
Gannan state (GN)	0.619	0.767	0.807	irs	Ineffectively
Average value	0.962	0.978	0.981		

#### 3.1.2 DEA-Malmquist results of the index model

The Malmquist dynamic evaluation results for the resource allocation efficiency of healthcare services at Gansu Provincial Hospital of TCM from 2018 to 2024 are shown in Table 7. The TFP is 0.955, showing a downward trend in overall productivity relative to the base year, with an average annual decline of 4.5% and overall operational efficiency remaining relatively low. The only period with a value above 1 was 2022–2023, at 1.319, indicating that overall efficiency improved during the study period. In other years, the TFP index was below 1.000, indicating overall low efficiency. The lowest TFP index was recorded in 2021-2022, at 0.811, indicating significant room for improvement. As shown in Table 7, the average value of the technical efficiency change index for the period 2018–2024 is 0.995, slightly below 1, indicating an overall positive trend. In the periods 2019–2020, 2022–2023, and 2023–2024, the EC values all exceeded 1; in the periods 2018–2019, 2020–2021, and 2021–2022, the technical efficiency change index was slightly below 1. Technical efficiency showed a fluctuating upward trend overall, with a slight decline during the period. The overall average TC value for the period 2018-2024 is 0.960, indicating significant room for improvement. In 2022-2023, the TC value is significantly above 1, reaching 1.318; in other years, the TC value remains below 1, indicating substantial potential for technological advancement.

**Table 7:** Malmquist Index of Efficiency of TCM Hospitals in Gansu, 2018–2023.

**Year**	**Technical efficiency change index (EC)**	**Technical change index (TC)**	**Pure technical efficiency change index (PECT)**	**Scale efficiency change index (SEC)**	**Total factor productivity (TFP)**
2018–2019	0.969	0.967	1.000	0.969	0.937
2019–2020	1.016	0.813	0.998	1.019	0.827
2020–2021	0.984	0.984	0.988	0.995	0.968
2021–2022	0.985	0.823	1.011	0.974	0.811
2022–2023	1.001	1.318	0.982	1.020	1.319
2023–2024	1.016	0.931	1.003	1.013	0.946
Average value	0.995	0.960	0.997	0.998	0.955

The Malmquist indices and their decompositions for TCM hospitals in different regions are shown in Table 8. Only the Malmquist index (i.e., TFP value) for Lanzhou is greater than 1, at 1.001. The TFP values for the other 13 regions are all declining, and the TC values for these 13 regions are all less than 1. The PECT values for most regions are 1.000, indicating that technical efficiency remains stable.

**Table 8:** Malmquist Index of Service Efficiency of TCM Hospitals in 14 CIties of Gansu, 2018–2024.

**Zones**	**Technical efficiency change index (EC)**	**Technical change index (TC)**	**Pure technical efficiency change index (PECT)**	**Scale efficiency change index (SEC)**	**Total factor productivity (TFP)**
LZ	1.003	0.999	1.000	1.003	1.001
JYG	1.000	0.998	1.000	1.000	0.998
JC	1.000	0.950	1.000	1.000	0.950
BY	0.994	0.912	0.996	0.998	0.906
TS	1.000	0.946	1.000	1.000	0.946
WW	1.029	0.961	1.028	1.001	0.989
ZY	0.996	0.974	1.000	0.996	0.970
PL	0.984	0.967	0.986	0.998	0.951
JQ	1.000	0.960	1.000	1.000	0.960
QY	1.010	0.977	1.010	1.000	0.988
DX	1.000	0.960	1.000	1.000	0.960
LN	1.000	0.929	1.000	1.000	0.929
LX	1.000	0.961	1.000	1.000	0.961
GN	0.921	0.945	0.941	0.979	0.870
Average value	0.995	0.960	0.997	0.998	0.955

### 3.2 Analysis results of HRAD

#### 3.2.1 Concentration of TCM hospital resources in various cities in Gansu in 2024

As shown in Table 9, the HRAD of TCM hospitals in various cities of Gansu range from 0.078 to 8.898, with significant disparities in the equity of regional distribution. The concentration indices of six regions—LZ, TS, PL, QY, DX, and LX—exceed 1, with LZ's HRAD values all above 3. The HRAD values of three regions—JYG, ZY, and GN —are all below 1, while some indicator groups in four cities—JC, BY, WW, and LN—are also below 1.

**Table 9:** Health Service Concentration of Traditional TCM Hospitals in 14 Zones of Gansu, 2024.

**Zones**	**TCM healthcare human resources**		**Number of TCM diagnostic and therapeutic devices (valued at over 5,000 yuan)**		**Number of TCM healthcare institutions**		**Number of TCM beds**		**PAD**
	**HRAD**	**Specific value**	**HRAD**	**Specific value**	**HRAD**	**Specific value**	**HRAD**	**Specific value**	
LZ	4.995	0.766	3.477	0.533	3.631	0.557	3.866	0.593	6.522
JYG	0.192	0.039	0.078	0.016	0.132	0.027	0.178	0.036	4.957
JC	0.955	1.105	0.566	0.655	1.118	1.295	0.524	0.607	0.864
BY	1.241	0.875	0.758	0.534	1.392	0.982	0.907	0.640	1.418
TC	3.537	0.908	2.491	0.640	4.758	1.222	3.868	0.993	3.894
WW	0.926	1.092	3.052	3.599	0.920	1.085	1.096	1.292	0.848
ZY	0.719	1.310	0.421	0.767	0.750	1.366	0.613	1.117	0.549
PL	3.789	1.221	2.921	0.941	4.373	1.409	3.722	1.199	3.103
JQ	0.128	1.223	0.148	1.414	0.131	1.257	0.106	1.014	0.104
QY	1.423	0.943	1.123	0.744	2.396	1.587	1.372	0.909	1.509
DX	2.419	0.995	1.865	0.767	2.053	0.845	3.363	1.384	2.431
LN	1.414	0.876	0.514	0.319	0.669	0.414	1.597	0.990	1.614
LX	3.010	0.608	8.898	1.798	2.150	0.435	5.223	1.056	4.949
GN	0.396	1.391	0.625	2.194	0.413	1.451	0.267	0.937	0.285

From the perspective of population distribution, the $\frac{HRAD_{\text{i}}}{PAD_{i}}$ ratio (hereinafter referred to as the ratio) of TCM hospitals in various cities of Gansu ranges from 0.016 to 3.599, indicating disparities in the equitable distribution of TCM hospital resources across regions based on population. In terms of the number of healthcare human resources, the ratios in WW, QY, and DX are closest to 1, indicating a relatively equitable distribution based on population. In terms of the number of medical devices, the ratio in PL is closest to 1, while WW has the highest ratio and JYG has the lowest ratio; the extreme values of all ratios are observed in the indicators. In terms of the number of healthcare institutions, BY and WW are close to 1, indicating a higher degree of population equity. In terms of the number of hospital beds, JC is close to 1, indicating better population equity, as shown in Table 9.

#### 3.2.2 HRAD of Gansu Provincial hospital of TCM from 2018 to 2023

As shown in Table 10, based on regional distribution, the HRAD values for medical resources at TCM hospitals ranged from 0.309 to 0.951 between 2018 and 2023. During the period from 2018 to 2023, the HRAD values for all indicators were below 1. From a population-based perspective, the $\frac{HRAD_{\text{i}}}{PAD_{i}}$ ratio for TCM hospitals in Gansu Province ranged from 0.298 to 0.948 from 2018 to 2023, with the overall ratios for all indicators showing a gradual increase year by year.

**Table 10:** HRAD Analysis of TCM Hospitals in Gansu Province, 2018–2023.

**Year**	**TCM healthcare human resources**		**Number of TCM diagnostic and therapeutic devices (valued at over 5,000 yuan)**		**Number of TCM healthcare institutions**		**Number of TCM beds**		**PAD**
	**HRAD**	**Specific value**	**HRAD**	**Specific value**	**HRAD**	**Specific value**	**HRAD**	**Specific value**	
2018	0.612	0.588	0.309	0.298	0.781	0.751	0.686	0.659	1.040
2019	0.620	0.578	0.479	0.447	0.636	0.593	0.773	0.720	1.073
2020	0.669	0.657	0.482	0.474	0.650	0.638	0.800	0.786	1.018
2021	0.713	0.704	0.584	0.577	0.676	0.667	0.829	0.818	1.013
2022	0.733	0.718	0.715	0.700	0.696	0.682	0.842	0.825	1.021
2023	0.823	0.821	0.769	0.767	0.666	0.664	0.951	0.948	1.003

### 3.3 Sensitivity and stability analysis

DEA model validity: Sensitivity and stability analysis results indicate that deleting multiple input and output variables has little effect on the overall efficiency score, confirming the internal robustness of the DEA model (Supplementary Table 1). Malmquist model validity: Statistical research shows that the efficiency estimates of SFA are not statistically different from the DEA results, which enhances the robustness of our research results (Supplementary Table 2).

## 4 Discussion

Compared to existing research, our study differs from previous macro-level approaches (such as those examining inter-state or inter-provincial comparisons) by focusing on a more specific examination of different cities within the same province. From the results of the scale returns analysis, ZY is found to be DEA-weakly efficient, with decreasing scale returns (drs), indicating that the city's output growth is lower than its input growth. This also suggests that the technical level and efficiency of traditional Chinese medicine hospitals in the region need to be improved. According to the evaluation criteria of DEA-related indicators, to improve efficiency while maintaining output levels, it is necessary to optimize input, establish a scientific and reasonable mechanism for allocating healthcare resources and improving efficiency, enhance technical efficiency, innovate hospital management systems and mechanisms, and optimize resource allocation. The three cities where the DEA is ineffective are BY, PL, and GN. Their scale returns are increasing (IRS), indicating that as production scale expands, decision-making units can achieve higher efficiency. Due to differences in resource allocation and the population served, there are significant variations in efficiency among different hospitals. Based on the results of the slack variable analysis, BY, PL, and GN all have multiple indicators that have not reached optimal levels. Considering the geographical location and development level of these three cities within Gansu Province, it can be inferred that there is an imbalance in resource allocation, specifically insufficient government investment in the healthcare sector, leading to shortages in human resources, equipment, and institutional infrastructure, which in turn results in lower service efficiency. Local governments need to improve resource allocation, alleviate disparities between regions with different economic levels, achieve effective allocation of resources in the health sector, and improve service efficiency.

From 2018 to 2024, the average TFP of Gansu Provincial Hospital of TCM was 0.955, with service efficiency showing a downward trend. Except for the years 2022 and 2023, when the TFP value exceeded 1, the TFP value decreased in all other years. This indicates that, under the same level of input, the output level and service efficiency still need to be improved. The TFP index grew between 2022 and 2023, presumably because cities strengthened their prevention and control measures and improved the efficiency of healthcare services after the COVID-19 pandemic had passed its peak. Among the TFP indices of cities in Gansu Province from 2018 to 2024, only Lanzhou has a TFP value greater than 1, while all other cities have TFP values less than 1. It should be noted that LZ, as the provincial capital of Gansu, has relatively abundant health resources. The local government needs to optimize infrastructure in underdeveloped areas, moderately increase resource investment, establish fair mechanisms, and improve the fairness of health resource allocation.

Comparing the HRAD data from 2018 to 2024, the geographical agglomeration degree and population agglomeration degree of healthcare services at Gansu Provincial Hospital of TCM were 0.309–0.951 and 0.298–0.948, respectively. It can be seen that during this period, geographical equity and population equity were relatively close, with the overall ratios of all indicators increasing year by year. However, by 2024, this balance was disrupted, with significant disparities in the distribution of geographical equity and population equity. In 2024, the ratios of geographical concentration and population concentration for service efficiency at Gansu Provincial TCM Hospital were 0.078–8.898 and 0.016–3.599, respectively, with population location equity far below geographical equity. Specifically, in 2024, the lowest scale efficiency of the Gansu Provincial TCM Hospital's service efficiency was 0.078, with a gap of 8.820 from the highest value, further highlighting the uneven distribution of service efficiency in geographical terms. In the process of improving the efficiency of TCM hospital services, it is necessary to focus on improving both geographical equity and population equity. Infrastructure upgrades and improvements should be carried out based on the economic levels of different regions to expand the geographical and population coverage of healthcare services, thereby improving the overall service level.

Our study has several advantages. First, by narrowing the scope of the study to individual cities, we have enriched the research findings on healthcare services at the micro level and effectively addressed the lack of research at this level. Second, combining the three-stage DEA model with the Malmquist model and HRAD analysis not only enables horizontal static measurement through the DEA model, but also enables vertical dynamic measurement across different years through the Malmquist model, reflecting trends in production efficiency over time. In addition, the HRAD analysis covers research on fairness, achieving innovation in research methods and strengthening the scope of the research. Third, the second stage of the DEA three-stage model involves constructing a SFA model that eliminates the influence of external factors such as environmental variables and random disturbances. Finally, this study provides valuable lessons and references for future research and other regions seeking to achieve high-quality and coordinated healthcare development.

## 5 Conclusion

Traditional Chinese Medicine (TCM) hospitals play a pivotal role in China's healthcare ecosystem, yet persistent inefficiencies and inequities in resource allocation undermine their capacity to deliver high-quality, fair care. Across Gansu Province's 14 cities (2018–2024), technical efficiency remained suboptimal despite minor improvements after accounting for environmental factors, while total factor productivity declined annually—indicating systemic room for enhancement. Geographically, resource distribution exhibited stark disparities, with low-efficiency regions disproportionately lacking infrastructure and personnel. Addressing these gaps requires targeted interventions.

In the subsequent sustainable development process, Gansu Province needs to improve technical efficiency and optimize internal resource allocation. This can be achieved by promoting benchmark management, strengthening capacity building, and introducing refined management to improve local technical efficiency. In addition, it is necessary to optimize geographical layout and promote equitable access to services. This can be achieved by implementing differentiated resource allocation, developing regional medical centers, and utilizing digital technology to bridge the gap between different regions. The findings of this study provide valuable empirical evidence for achieving sustainable development and providing equitable healthcare services, not only in China but also as a reference for other countries worldwide.

This study still has areas that require improvement. Assessing the efficiency and equity of healthcare resource allocation requires careful selection of variables aligned with institutional characteristics while ensuring data availability to measure core competencies. Although quantitative variables such as TCM healthcare human resources and Number of TCM beds reveal fundamental resource distribution patterns, they remain limited in comprehensive performance evaluations. Relying solely on quantitative indicators risks overlooking critical qualitative factors, thereby compromising research outcomes. Qualitative variables exert significant influence on resource allocation efficiency and equity. Institutional culture shapes staff behavior, while patient satisfaction reflects service quality. Though difficult to quantify, these qualitative factors substantially impact resource allocation efficiency and equity within healthcare services. In subsequent research, evaluation methods integrating quantitative and qualitative indicators can provide more comprehensive insights into the efficiency and fairness of healthcare resource allocation. This combined qualitative-quantitative approach enables more accurate assessments, thereby supporting sustainable healthcare development.

## Data Availability

Publicly available datasets were analyzed in this study. This data can be found here: https://tjj.gansu.gov.cn/tjj/index.shtml.
