# Supplementary material for: Efficiency and equity of resource allocation in healthcare services using DEA and concentration indices: evidence from the traditional medicine hospital in Gansu Province, China
Source: Front Public Health. 2025 Oct 1;13:1674348. doi: 10.3389/fpubh.2025.1674348 (PMC12521092; doi:10.3389/fpubh.2025.1674348)
Supplement: Supplementary file 1 [file Table_1.docx]

| **Variables/removed** | **Efficiency average scores** | **Spearman rank correlation(sig)** | **Wilcoxon p value** |
| --- | --- | --- | --- |
| TCM healthcare human resources | 0.956 | 0.853(＜0.001) | 0.317 |
| Number of TCM diagnostic and therapeutic devices (more than 5000 yuan) | 0.956 | 0.993(＜0.001) | 0.180 |
| Number of TCM healthcare institutions | 0.960 | 1.000(0.000) | 0.157 |
| Number of TCM beds | 0.888 | 0.700(0.005) | 0.018 |
