# Supplementary material for: Efficiency and equity of resource allocation in healthcare services using DEA and concentration indices: evidence from the traditional medicine hospital in Gansu Province, China
Source: Front Public Health. 2025 Oct 1;13:1674348. doi: 10.3389/fpubh.2025.1674348 (PMC12521092; doi:10.3389/fpubh.2025.1674348)
Supplement: Supplementary file 2 [file Table_2.docx]

| City | DEA | SFA | Spearman rank correlation(sig) | Wilcoxon p value |
| --- | --- | --- | --- | --- |
| LZ | 1.000 | 1.000 | 0.791 (＜0.001) | 0.686 |
| JYG | 1.000 | 1.000 |  |  |
| JC | 1.000 | 0.988 |  |  |
| BY | 0.962 | 0.956 |  |  |
| TS | 1.000 | 1.000 |  |  |
| WW | 1.000 | 1.000 |  |  |
| ZY | 0.975 | 1.000 |  |  |
| PL | 0.908 | 0.911 |  |  |
| JQ | 1.000 | 1.000 |  |  |
| QY | 1.000 | 1.000 |  |  |
| DX | 1.000 | 1.000 |  |  |
| LN | 1.000 | 1.000 |  |  |
| LX | 1.000 | 1.000 |  |  |
| GN | 0.610 | 0.619 |  |  |
